# Granulation Methods and the Mechanisms for Improving Hardness of Loxoprofen Sodium Hydrate-Containing Tablets

**DOI:** 10.3390/pharmaceutics17040455

**Published:** 2025-04-01

**Authors:** Aya Kuwata, Agata Ishikawa, Tetsuo Ono, Etsuo Yonemochi

**Affiliations:** 1 Research & Development Headquarters Self-Medication, Taisho Pharmaceutical Co., Ltd., 1-403 Yoshino-Cho, Kita-ku, Saitama 331-9530, Japan; 2 Department of Physical Chemistry, Hoshi University, 2-4-41 Ebara, Tokyo 142-0063, Japan; 3 Department of Physical Chemistry, International University of Health and Welfare, 4-3 Kozunomori, Chiba 286-8686, Japan

**Keywords:** loxoprofen, tensile strength, hardness, tablet, surface free energy, hydrate, crystal structure

## Abstract

**Objectives:** We investigated the compression mechanisms for loxoprofen sodium (LXP), which is known to occur as a dihydrate, and identified parameters that influence the tablet hardness of LXP tablets prepared by the wet granulation method. **Method:** LXP granules were prepared with water or ethanol as the solvent, dried under various conditions and sieved for particle size control, with 1% Mg-st added before tablet compression. **Results**: The findings indicated that both the granulation solvent and drying temperature significantly impacted the tablet hardness. Granules prepared with ethanol exhibited higher hardness as compared with those prepared with water. The tablet hardness varied with varying drying temperatures. **Discussion:** Principal component analysis (PCA) identified positive correlations between the tablet hardness and the surface free energy (SFE), polar component (γ(p)), and cohesion, and a negative correlation with the dispersive component (γ(d)). Granules prepared with ethanol exhibited a higher γ(p), likely due to the differing solubility in ethanol and water, leading to enhanced interparticle binding. This study confirmed that use of the eutectic mixture of LXP and Mg-st exerted no significant influence. Crystal structure analysis indicated that the hydration states varied according to the drying temperature, suggesting the higher γ(p) in anhydrous forms, due to the lower hydrophobicity, contributed to increased tablet hardness. **Conclusion:** This research offers insights for optimizing the formulation conditions to improve the LXP tablet hardness. Appropriate selection of the solvent and drying temperature mitigates tablet hardness issues, while assessment of SFE can help in the selection of suitable additives.

## 1. Introduction

Loxoprofen sodium (LXP) is a prodrug type of pharmaceutical. It is a non-steroidal anti-inflammatory drug (NSAID) of the 2-phenylpropionate class and is sold in at least 28 countries [1]. In Japan, LXP is the most frequently prescribed NSAID, being prescribed to 32.5% of patients [2]. It is widely used for the treatment of osteoarthritis, adhesive capsulitis, rheumatoid arthritis, arthritis, dental pain, and postoperative pain [3]. In addition to oral formulations such as tablets and granules, various dosage forms including gels, patches, and ointments are also available. In Japan, LXP was switched to being sold as an over-the-counter drug in 2011 and to being one of the first-choice analgesics.

Furthermore, research into LXP formulations is ongoing [4]. Antipyretic and analgesic drugs other than LXP, such as ibuprofen (IB) and acetaminophen (APAP), have been used for a long time in Japan. However, LXP differs significantly from IB and APAP. For example, both IB and APAP exist in the free form and show low solubility in water [5]. In contrast, LXP is a salt, has high solubility in water, and exhibits viscosity upon dissolution, resulting in poor disintegration characteristics. Furthermore, IB and APAP are needle-shaped crystals, which are associated with poor compressibility and various tableting issues such as capping [6,7]. Additionally, IB has a low melting point, which has been reported to cause adhesion problems [8]. On the other hand, there are few reports on the compressibility of LXP.

LXP is a dihydrate, and its hydration level significantly influences the manufacturing process. For instance, it can easily dehydrate under low-humidity conditions or when heated [9]. The dehydration of hydrates alters their physical and chemical properties, which can have a considerable impact on their manufacture [10,11]. Therefore, it is essential to establish appropriate temperature and humidity conditions during the manufacturing process [12]. Furthermore, hydrates generally tend to form hydrogen bonds with other molecules, which is known to impact the tablet hardness [13]. To ensure consistent product quality and efficacy in pharmaceuticals, optimizing the formulation process is essential [14]. Understanding how manufacturing conditions affect the physical properties of tablets is crucial for this optimization. Various methods for tablet manufacturing are employed, with the wet granulation method being one of the most commonly used. Wet granulation involves adding a solvent to a powder and includes a drying process. Granules obtained by wet granulation are characteristically close to being spherical and exhibit a wide particle size distribution [15]. Thus, wet granulation is chosen for cases where ensuring content uniformity proves challenging due to segregation, when flowability is poor, or when the compression property is low. On the other hand, use of a solvent and drying of the wet granules are essential in the wet granulation process. Therefore, establishing drying conditions based on the stability of the drug and the solvent used, as well as the melting point of the drug, is critical [15,16,17]. Therefore, there are concerns that the physical properties of LXP may vary depending on the manufacturing conditions. Thus, elucidating the mechanisms of LXP’s compressibility is crucial for advancing its formulation. In this study, we investigated the impact of the solvent types and drying temperature on the properties of tablets produced from wet granulation of LXP. Our findings indicate that the tablet hardness varies with different solvents and temperature conditions. Furthermore, we analyzed the formulations produced, identified the physicochemical characteristics related to changes in the tablet properties due to variations in the solvents and drying temperatures, and elucidated the mechanisms involved in tablet hardness. This research is expected to facilitate the optimization of manufacturing processes and ensure production of high-quality pharmaceuticals for patients.

## 2. Materials and Methods

### 2.1. Materials

We used “Loxoprofen Sodium Dihydrate powder grade” manufactured by Daiwa Pharmaceutical Co., Ltd. (Toyama-shi, Toyama, Japan). as LXP. We used “Purified Water Yoshida” conforming to the Japanese Pharmacopoeia sourced from Yoshida Pharmaceutical Co., Ltd. (Chuo-ku, Tokyo, Japan). and “Ethanol 95” conforming to the Japanese Pharmacopoeia procured from Nippon Alcohol Sales Co., Ltd. (Chuo-ku, Tokyo, Japan). We also used “Magnesium stearate Light grade” conforming to the Japanese Pharmacopoeia from Taihei Chemical Industry Co., Ltd. (Osaka-shi, Osaka, Japan).

### 2.2. Preparation of Granules

LXP (100%, *w*/*w*) was thoroughly mixed in a mortar until there were no agglomerates. Various solvents (10%, *v*/*v*) were added to the mixed LXP and the mixture was kneaded for 5 min. The resulting wet granules were dried under various temperature conditions for 24 h to remove the solvent and obtain dry granules. The obtained dry granules were then sieved, and granules classified between 106 μm and 180 μm were used for examination (Table 1).

### 2.3. Particle Size Distributions

Approximately 5 g of each granule was analyzed using a particle size distribution measurement device (Robot Shifter RPS-01, Seishin Enterprise Co., Ltd., Chiyoda-ku, Tokyo, Japan). Sieve mesh sizes of 710, 500, 355, 250, 180, 150, 106, and 75 μm were employed, and the samples were vibrated for 3 min with Level 4 vibration and a pulse interval of 1 s (*n* = 1). The relative width of the particle size distribution (*Rw*) was calculated using the following equation:

$$R_{W}=\frac{D_{90}-D_{10}}{D_{50}}$$

where *D*_10_, *D*_50_, and *D*_90_ are 10, 50, and 90%, respectively, of the accumulated particle size on a screen.

### 2.4. Scanning Electron Microscopy (SEM)

Particle surfaces were examined using a scanning electron microscope (SEM) (TM-1000 Tabletop Scanning Electron Microscope, Hitachi High Technologies, Minato-ku, Tokyo, Japan) which could be operated without the need for metal deposition. Samples were placed on a plate without being coated with Au before imaging. An accelerating voltage was set to 5 kV.

### 2.5. Flow Properties

The flow properties of the granulated particles were measured using an FT4 powder rheometer (Freeman Technology, Tewkesbury, UK) [18,19,20]. To achieve highly reproducible results, FT4 conditioned the powder before measurement. The conditioning was performed using a blade that moved at a specified helix angle and tip speed, resulting in a uniform and highly reproducible state. Approximately 70 g of each batch of granules were filled into an 85 mL measuring container (*n* = 1). A 48 mm blade was used to determine the total energy required for the downward test on the conditioned powder, as well as the conditioned bulk density (CBD) and basic flow energy (BFE).

### 2.6. Shear Force

The shear stress was measured using a shear force kit of the FT4 powder rheometer (Freeman Technology, Tewkesbury, UK). To achieve highly reproducible results, FT4 conditioned the powder before measurement. The conditioning was performed using a blade that moved at a specified helix angle and tip speed, resulting in a uniform and highly reproducible state. The constant load shear testing method involved shearing the powder layer while applying a constant vertical stress on the upper surface of the powder layer, allowing for the relationship between vertical stress and shear stress to be obtained (*n* = 1). This method was used to calculate the angle of internal friction (AIF), flow function (FF), and cohesion.

### 2.7. Surface Free Energy

The surface tension of each sample (20 ± 0.5 °C) was measured using the capillary method with a tensiometer (Tensiio, KRUSS, KRÜSS GmbH, Hamburg, Germany). A fixed amount of the sample packed in a glass tube was immersed in solutions with known surface free energy (SFE) values—hexane, tetrachloromethane, 1-chlorobutane, and 1-nitropropane—at room temperature (20 ± 0.5 °C). The point of liquid contact with the powder was designated as zero for measuring the contact angle. Each measurement was repeated five times and the average value obtained (*n* = 5). The contact angles were analyzed using the Owens–Wendt–Rabel–Kaelble equation to calculate the polar component of the solid’s SFE, γ(p), and γ(d) [21,22].

$$\gamma L\times \left(1+\cos\theta \right)=2\sqrt{\gamma \left(d\right)\;\gamma L(d)}+2\sqrt{\gamma \left(p\right)\;\gamma L(p)}$$

In the above equation, *θ* signifies the contact angle, while γL and γ represent the SFE of the liquid and solid, respectively. The variables p and d indicate the polar and dispersive components of the SFE, respectively.

### 2.8. Moisture Content

The moisture content of each sample was measured using a heated drying moisture analyzer (MX-50, A&D). Approximately 5 g of each sample was placed in a stainless steel weighing pan and heated at 105 °C for 30 min to determine the moisture content (Loss on Drying, LOD). Each measurement was repeated three times and the average values obtained (*n* = 1).

### 2.9. Thermogravimetric Analysis

Differential scanning calorimetry (DSC) was performed using a differential scanning calorimeter (Thermo plus EVO2 DSCvesta, Rigaku, Shibuya-ku, Tokyo, Japan). Approximately 5 mg of each sample was accurately weighed in an aluminum pan. The sample pan was heated from 30 °C to 250 °C at a rate of 5 °C per minute, using an empty aluminum pan as reference. Closed pans were utilized for the DSC measurements. We used each LXP sample excluding Mg-st for the measurements. Thermogravimetric analysis was carried out using a Thermogravimetric Analyzer (Thermo plus EVO2 TGDTA8122, Rigaku, Shibuya-ku, Tokyo, Japan). The sample pan was heated from 30 °C to 250 °C at a rate of 5 °C per minute, using an empty aluminum pan as reference.

### 2.10. Powder X-Ray Diffraction Measurement

Each granulated sample was analyzed using a powder X-ray diffractometer (SmartLab, Rigaku, Shibuya-ku, Tokyo, Japan). Measurements were conducted with Cu Kα radiation at 40 kV and 30 mA, over a scanning range of 0° to 50°, with a scan rate of 0.5° per minute.

### 2.11. Preparation of Tablets

Tablets were obtained through compression molding using a single-punch tablet press (HANDTAB, Ichihashiseiki Co., Ltd., Kyoto-shi, Kyoto, Japan). The granules were mixed to achieve a composition of 99.0% (*w*/*w*) LXP and 1.0% (*w*/*w*) Mg-st, resulting in a homogeneous mixture.

The reason for adding 1% Mg-st is that when tablets were pressed using only LXP, adhesion occurred, making it impossible to form tablets. Therefore, we decided to add a minimum of 1% Mg-st to enable tablet formation. The mixture of granulated particles and Mg-st were passed through a sieve with a mesh opening of 710 μm, then placed in a plastic bag and mixed for 3 min. This mixture was then compressed into tablets with a target mass of approximately 250 mg using an 8 mm flat-faced punch at compression pressures of 5 kN and 10 kN.

### 2.12. Evaluation of Tablets

The tablet hardness was measured using a tablet hardness tester (Schleuniger 8M tester, Pharmatron Dr. Schleuniger, Thun, Switzerland) by applying vertical compression at a rate of 0.2 mm/sec to determine the breaking strength, which is the force required to fracture the tablet (*n* = 3). The tensile strength (TS) was then calculated using the following formulation based on the obtained results. The tensile strength is considered as an indicator of the tablet hardness. Measurements were performed in triplicate for each sample, and the average value was determined. Results are expressed as the mean ± standard deviation of three independent experiments.

$$Tensile\;strength=\frac{2F}{\pi Dt}$$

*F*: Tablet hardness [N]; *D*: Tablet diameter [mm]; *t*: Tablet thickness [mm].

### 2.13. Statistical Analysis

Two-way ANOVA was performed on the data of TS. The data analysis was con-ducted using JMP (version 18, SAS Institute Inc., Cary, NC, USA). Differences in solution (X1) and dry temperature (X2) were used as explanatory variables for this analysis, and *p*-values of <0.05, 0.01, or 0.001 were considered statistically significant. Furthermore, Tukey’s test was conducted to compare the differences in TS between each sample, and *p*-values of <0.05, 0.01, or 0.001 were considered statistically significant.

Principal component analysis (PCA) was conducted using JMP (version 18, SAS Institute Inc., Cary, NC, USA) on the data for BFE, CBD, Cohesion, FF, AIF, γ(p), γ(d), SFE, TS at 5 kN and TS at 10 kN. The data used for the PCA were standardized [23]. Additionally, correlation analysis of the average values of the data was performed using the JMP software.

## 3. Results

### 3.1. Preparaion of Granules Under Different Granulation Conditions

**Figure 1:**
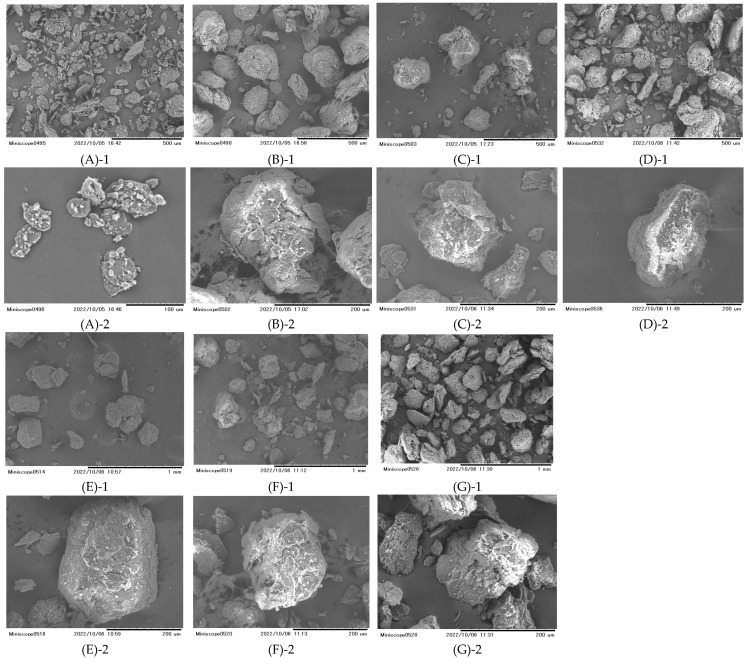
Particle shapes: (**A**) Original, (**B**) 1-1, (**C**) 1-2, (**D**) 1-3, (**E**) 2-1, (**F**) 2-2, (**G**) 2-3. Branch No. 1; a magnification of ×150, Branch No. 2: a magnification of ×400. (**A**)-2; a magnification of ×600.

Granules of LXP were manufactured under various granulation conditions (Table 1). Particle size is one of the factors that affects tablet hardness and in this study, to identify factors other than particle size that are known to affect tablet hardness, we adjusted the particle size distribution to be as similar as possible. Granules of each sample classified between 106 μm and 180 μm were used. The particle size distributions are presented in Table 2 and the particle shapes of the obtained granules are shown in Figure 1. As a result, the particle size distribution could be adjusted to a similar degree for all granules (1-1~2-3). The original powder exhibited aggregation of plate-like crystals. In contrast, irrespective of the granulation solvent and drying temperature, all the granulated particles were nearly spherical in shape, and the particle surface was observed to consist of LXP particles from the original powder in a dissolved state.

### 3.2. Tabletability of Granules of Each Sample Under Different Granulation Conditions

**Figure 2:**
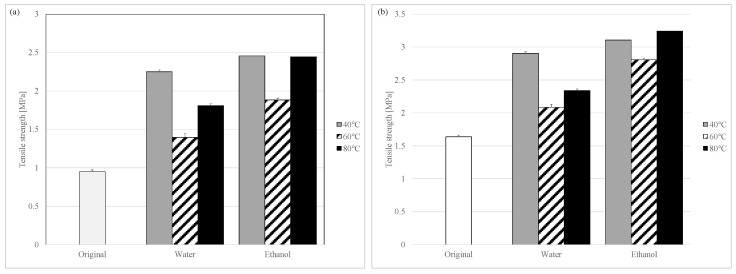
Tensile strength with different granulation solvents and drying temperatures of each LXP sample: compaction pressure: (**a**) 5 kN, (**b**) 10 kN. Each bar indicates the average ± S.D. (*n* = 3).

Granules of each sample under different granulation conditions mixed with magnesium stearate (Mg-st) were compressed into tablets, and their tensile strength was calculated (Figure 2). The significant differences in the tensile strength of each sample are shown in Table 3, Table 4 and Table S1. The effects of differences in the tablet compression pressure were minimal. IB and APAP, known for their low compressibility, cannot be used in their original powder form with Mg-st 1% for preparing tablets, however, tablets of LXP could be prepared using the original powder with Mg-st 1%. The original powder was plate-like crystalline (Figure 1). Plate-like crystals have fewer contact points and a smaller contact area among particles, leading to poor flow properties, increased void formation, and poor packing characteristics, which are known to negatively affect the compressibility and result in lower tablet hardness as compared to spherical granules [24]. Additionally, granules produced by high-shear wet granulation show increased density, which enhances the strength of the granules and improves the tablet hardness compared with the original powder [25]. Therefore, it is inferred that LXP granules also show improved hardness due to wet granulation as compared with the original powder. In regard to the solvents used for granulation, ethanol-granules (2-1 to 2-3) showed higher hardness than water-granules (1-1 to 1-3) at the same drying temperature. When examining drying temperatures, tablets dried at 40 °C, 60 °C, and 80 °C exhibited varying hardness values, with 60 °C yielding the lowest hardness. This trend was consistent across granules produced with both ethanol and water. In this study, the impact of the particle size was minimized. The results in Figure 2 indicated little variation in particle shape among the granulated samples. Consequently, further evaluation of the granule characteristics was performed to explore additional factors influencing the tablet hardness related to the granulation conditions, specifically the choice of solvent and the drying temperature.

## 4. Discussion

### 4.1. Identification of Granule Properties Affecting the Hardness of LXP-Containing Tablets Manufactured Under Various Conditions

#### 4.1.1. Principal Component Analysis

**Figure 3:**
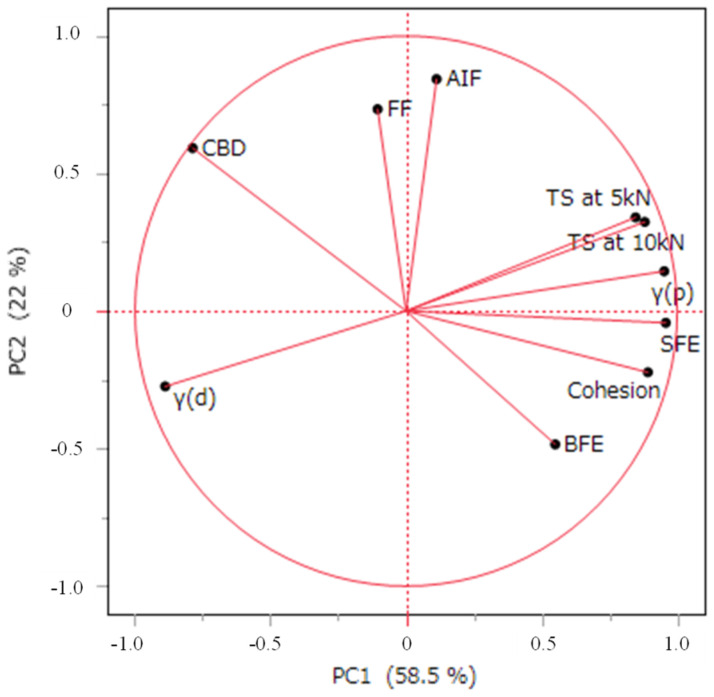
Principal component analysis: loading plot.

To visualize the multidimensional properties of LXP granules produced under different manufacturing conditions, we evaluated parameters related to flowability, shear testing, and SFE. Principal component analysis (PCA) was performed using these granule parameters along with the tensile strength results [23,26]. The granule parameters for each sample used in the analysis are summarized in Table 5. The first principal component (PC1) explained 58.5% of the variance, while the second principal component (PC2) explained 22.0%, together accounting for 80.5% of the dataset. The loading plot is illustrated in Figure 3. Comparing the vectors of each characteristic in the loading plot allows us to understand the relationships among them. Vectors pointing in the same direction indicate a positive correlation, whereas vectors pointing in opposite directions suggest a negative correlation. As indicated in Figure 3, the tensile strength, the polar component γ(p), SFE, and cohesion are aligned in the positive direction along the *x*-axis, while the dispersive component γ(d) is pointing in the negative direction. This suggests that PC1 reflects the interparticle bonding strength linked to the surface state of the particles. Therefore, different LXP granules with varying interparticle bonding strengths were obtained by manipulating the solvent type and drying temperature during the wet granulation process. PC2 showed a close relationship with the FF and AIF derived from shear testing. Table 6 provides the correlation matrix for the LX granule characteristics. A strong correlation (|r| > 0.70) was noted among the tensile strength, SFE, dispersive component, polar component, SFE, and cohesion, aligning with the findings from the loading plot.

#### 4.1.2. Effect of Surface Free Energy on Tablet Hardness

**Figure 4:**
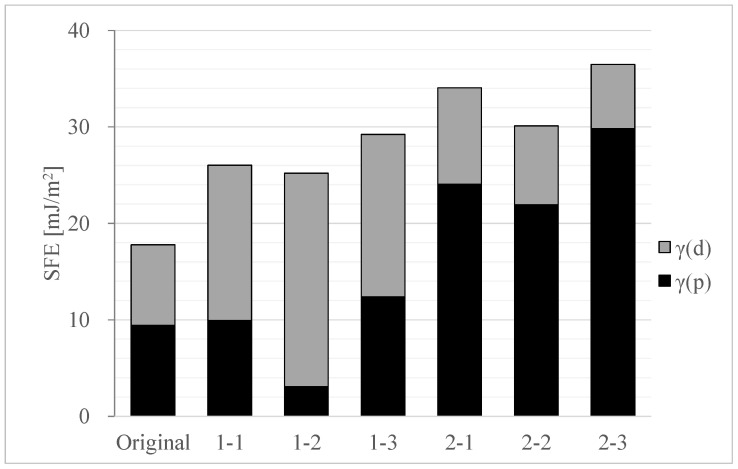
Comparison of γ(d) and γ(p) of SFE obtained with different granulation solvents and drying temperatures for each LXP sample; 1-1–1-3: Water, 2-1–2-3: Ethanol, Branch No.1: 40 °C, Branch No.2: 60 °C, Branch No.3: 80 °C.

We examined the details of the SFE for each sample. The results are shown in Figure 4. The original powder exhibited a lower SFE as compared with the granules, and the ratio of γ(p) to γ(d) was similar. It has been reported that higher SFE values correlate with increased tablet hardness [21,27,28,29,30]. Additionally, it has been documented that the state of granulation affects the SFE [31,32]. The analysis results also indicated that the adhesion strength influences the tablet hardness. Therefore, it was inferred that the increased hardness of the granulated tablets resulted from increased SFE during granulation, leading to enhanced inter-particle adhesion. We also examined the effects of the granulation solvent. Water-granulated samples (1-1 to 1-3) exhibited high γ(d) values, negatively correlating with the tensile strength, while ethanol-granulated samples (2-1 to 2-3) showed high γ(p) values, being positively correlated with the tensile strength. An increase in γ(p) strengthens the attractive forces among particles, enhancing the bonding strength and interactions among polar molecules [21,33].

Polar molecule interactions include hydrogen bonding and dipole–dipole interactions [34,35]. Additionally, it has been reported that differences in hydroxyl groups lead to variations in γ(p), which in turn results in differences in hardness [36].

LXP has different solubility in the solvents used in this experiment, namely water and ethanol [37]. SEM images of Figure 1 reveal that drug dissolution occurred in the solvent during wet granulation.

Thus, the reason for the increased γ(p) and higher hardness when granulating LXP with ethanol is that variations in the solubility of LXP during granulation lead to differences in the surface states of the granulated particles, which in turn influence γ(p) and enhance tablet hardness.

The increase in γ(d) is reported to enhance van der Waals forces; however, these forces are weaker than polar interactions [38,39]. Therefore, the increase in γ(d) is expected to result in a smaller increase in tablet hardness compared to the increase in γ(p). Consequently, it was anticipated that samples 1-1 to 1-3 produced via water granulation, which exhibited an increase in γ(d), would have lower hardness than samples 2-1 to 2-3 obtained through ethanol granulation, where γ(p) was elevated.

The granulation solvent influences the ratio of γ(p) to γ(d), and it has been confirmed that γ(p) increases with the use of ethanol as the solvent for LXP. Additionally, we explored the impact of drying temperature. For all samples granulated with each solvent, the SFE of the granulated particles differed with the drying temperature used. It is reported that SFE changes with temperature or hydration state [28]. Given that LXP is a dihydrate, its hydration state may vary with drying temperature [40,41]. Therefore, it is inferred that the hydration state of LXP changes with the drying temperature, consequently affecting the SFE.

In conclusion, the surface state of LXP is influenced by the granulation solvent and the drying temperature used, and high values of SFE and enhanced inter-particle adhesion can be obtained, with improved tablet hardness. Increase in the γ(p) was found to significantly influence tablet hardness, with ethanol granulation boosting γ(p) and water granulation enhancing γ(d).

### 4.2. Morphological Study

#### 4.2.1. Moisture Contents of Samples

It has been reported that approximately one-third of pharmaceutical compounds have the potential to form crystalline hydrates [13,42]. Moreover, hydrates are known to easily form hydrogen bonds with other molecules due to water molecules, affecting the tablet hardness [43]. In addition, it has been reported that the state of hydration influences the SFE [28]. Therefore, the intrinsic properties of LXP in each of the granulated particle types were evaluated [44]. The results of measurement of the loss on drying (LOD) for each granule are presented in Table 7. The original powder, which is a dihydrate, exhibited an LOD of 12.02%, which is consistent with the theoretical value for dihydrates. The samples 1-1 and 2-1 dried at 40 °C showed LOD values of 6.29% and 6.23%, respectively, which is equal to or very close to the theoretical weight loss rate of 6.29% for LXP monohydrate. Thus, our findings suggested that the granules dried at 40 °C for both 1-1 and 2-1 may consist mainly of monohydrates due to dehydration. The LOD for 1-2 and 2-2 dried at 60 °C was around 0.5%, while 1-3 and 2-3 dried at 80 °C showed an LOD of nearly 0.3% or lower. No significant difference in the LOD was observed depending on the solvent used. In general, dihydrates are reported to dehydrate stepwise, transitioning through monohydrate to anhydrous forms [45]. This suggests that the granules dried at 60 °C mainly consisted of anhydrous material with a small amount of monohydrate, while the granules dried at 80 °C were likely in an anhydrous state.

#### 4.2.2. Thermal Analysis

**Figure 5:**
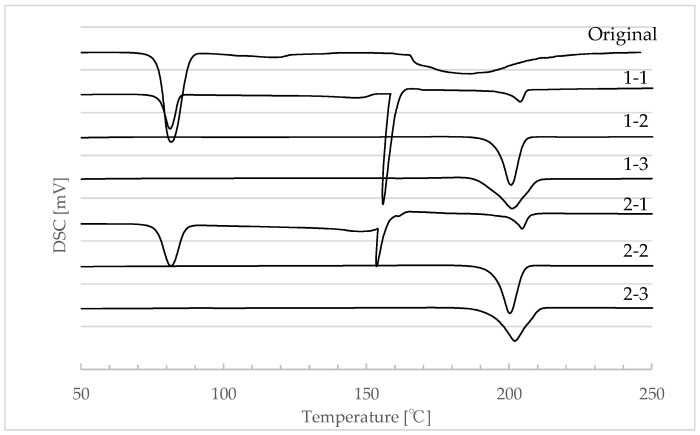
Differential scanning calorimetry (DSC) of each LXP sample. 1-1: Water-40 °C, 1-2: Water-60 °C, 1-3: Water-80 °C, 2-1: Ethanol-40 °C, 2-2: Ethanol-60 °C, 2-3: Ethanol-80 °C.

To further investigate the state of hydration of each sample, thermal analysis was conducted using differential scanning calorimetry (DSC). Measurements were conducted on samples without the addition of Mg-st. DSC has been reported to be useful for confirming hydration states [44,46]. The results are shown in Figure 5. The original powder exhibited endothermic peaks at approximately 85 °C, 123 °C, and 190 °C. No significant differences due to the granulation solvent were observed in the DSC. In regard to the drying temperature, use of 40 °C yielded a different DSC behavior as compared with that at 60 °C or 80 °C. The granules obtained from drying at 40 °C (samples 1-1 and 2-1) showed endothermic peaks at around 85 °C and 190 °C, similar to the original powder, with an additional endothermic peak near 154 °C. In contrast, granulated granules dried at 60 °C and 80 °C (samples 1-2, 1-3, 2-2, and 2-3) exhibited only the endothermic peak at approximately 190 °C. The peak near 190 °C is believed to be related to decomposition. It was inferred that the samples dried at 60 °C and 80 °C were in an anhydrous state (Table 7). A weight loss was observed around 85 °C and 123 °C using TG/DTA. Therefore, the peak near 85 °C, present only in the original and the granules dried at 40 °C, and near 123 °C, was suspected to be related to dehydration. These findings suggest that the granules dried at 40 °C may be monohydrates [46].

In this study, Mg-st is blended with LXP granules during tablet compression. The reason for this is that when pressing tablets using only LXP, adhesion occurred, making it impossible to form tablets. Mg-st improves the flowability of the powder and helps prevent adhesion. However, excessive addition can reduce tablet hardness. Additionally, due to its hydrophobic nature, magnesium stearate can lead to delays in disintegration time. Therefore, we decided to add a minimum amount of 1% Mg-st, which allows for compression of tablets. Eutectic mixtures have been reported to contribute to increased tablet hardness [47,48]. Additionally, ibuprofen, a nonsteroidal anti-inflammatory drug (NSAID), shows a melting point depression when mixed with Mg-st [49]. Therefore, the mixture of LXP and Mg-st was also examined using DSC. However, no melting point depression was observed. This may be attributed to the low concentration of Mg-st used in this study, which is only 1%, and the mixing method which needs minimal shear stress. Therefore, it was concluded that there is no significant influence from the eutectic mixture in this research.

#### 4.2.3. Powder X-Ray Diffraction Measurement

**Figure 6:**
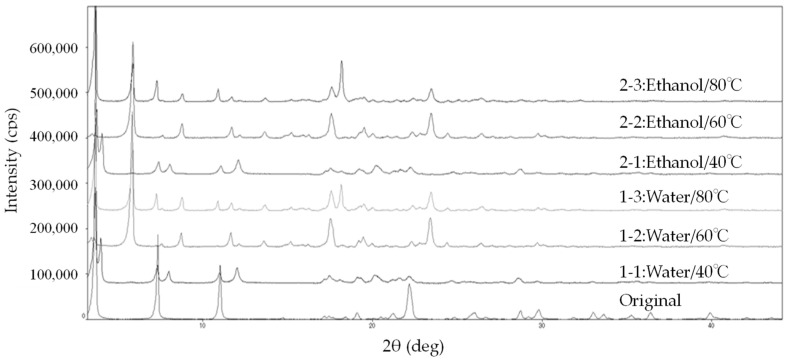
X-ray powder profiles with different granulation solvents and drying temperatures of each LXP sample.

The previous results suggested that the state of hydration varies with the drying temperature; thus, we analyzed the powder X-ray diffraction (PXRD) of each granulated sample (Figure 6). PXRD is a widely used technique for studying crystal structures and can be applied to solid powders. It is particularly effective for distinguishing between hydrates and anhydrous forms [50]. Reports indicate that diffraction patterns differ dramatically between hydrates and dehydrated materials [51,52,53,54]. The absence of halo patterns in all samples indicates high crystallinity. The diffraction pattern of the original sample exhibited characteristic peaks at 6°, 16.5°, 19.5°, and 22°. Granules dried at 40 °C (1-1 and 2-1) exhibited additional specific peaks at 7° and 12°, while those dried at 60 °C (1-2 and 2-2) showed specific peaks at 8°, 11.5°, 18°, and 24°. Granules dried at 80 °C (1-3 and 2-3) showed another specific peak at 18.5°. No changes in crystal structure due to different granulation solvents were observed. Thus, four distinct diffraction patterns were confirmed: Original, 40 °C dried (1-1, 2-1), 60 °C dried (1-2, 2-2), and 80 °C dried (1-3, 2-3). These findings indicate that the crystal structure of LXP changes upon wet granulation, and the granules dried at different temperatures (40 °C, 60 °C, and 80 °C) exhibit distinct crystal structures. Consequently, the results shown in Table 7 and Figure 5 show that LXP in the granulated particles exists in different hydration states based on the drying temperatures used during the granulation process. It is suggested that variations in the hydration states among granulated particles at different drying temperatures influence the differences in the SFE values [52,53].

The granules dried at 40 °C (1-1 and 2-1) are considered to be monohydrates (Table 7, Figure 5). It has been reported that hydrates exhibit higher tablet hardness compared to anhydrates [48]. Therefore, in the case of the 40 °C dried granules, the increase in hardness is suggested to be due not only to SFE effects but also to hydrates. Additionally, the granules dried at 60 °C (1-2 and 2-2) and 80 °C (1-3 and 2-3) are thought to be anhydrates with different crystal structures. It has been reported that crystal structure influences SFE [55,56]. The differences in SFE due to the distinct crystal structures of the 60 °C and 80 °C dried granules are considered to have affected tablet hardness. It is generally known that hydrates are more hydrophobic than anhydrates [57]. Additionally, a lower γ(p) is associated with lower tablet hardness [21,33]. Therefore, we considered that the anhydrate has low hydrophobicity, resulting in a higher γ(p), which contributed to the increase in tablet hardness.

## 5. Conclusions

LXP granules were prepared by a wet granulation process under various manufacturing conditions to evaluate the effects on the tablet properties. The hardness of the tablets increased with the granulation, with granules produced using ethanol as a solvent exhibiting greater hardness than those produced with water. Additionally, the tablet hardness varied with the drying temperature. Thus, the granulation conditions were confirmed to exert an impact on the hardness of tablets containing LXP. PCA showed a positive correlation between tablet hardness and SFE, γ(p), and adhesion strength.

In this study, we identified parameters affecting the tablet hardness of LXP-containing tablets. A detailed investigation of the mechanisms revealed that the increase in γ(p) value associated with the use of ethanol as the granulation solvent can be attributed to differences in the particle surface state caused by variations in the solubility of LXP in water and ethanol. We also confirmed that the γ(p) value was influenced by the hydration state of the granules, which was affected by the drying temperature. According to these results, we suggest that controlling the hydration state allows control of the tab-let hardness, serving as valuable information for optimizing the formulation design. For example, in the formulation of active ingredients prone to hardness reduction, evaluating the surface free energy composition facilitates the selection of suitable excipients to enhance the hardness. Future research must focus on identifying effective excipients based on surface free energy considerations. Moreover, our findings indicate that the surface free energy of LXP varies with manufacturing conditions, including choice of the solvent and drying temperature, underscoring the relevance of surface free energy in selecting optimal manufacturing conditions. Thus, this research pro-vides insights for provides insights for both optimization of the formulation design and the manufacturing parameters for LXP tablets.

## Figures and Tables

**Table 1:** Granulation conditions.

Samples	Solution	Dry Temperature [°C]
Original	-	-
1-1	water	40
1-2	water	60
1-3	water	80
2-1	ethanol	40
2-2	ethanol	60
2-3	ethanol	80

**Table 2:** Particle size distributions of samples.

Sample	Original	1-1	1-2	1-3	2-1	2-2	2-3
D10 [μm]	20.9	132.3	126.6	122.8	130.4	122.4	129.1
D50 [μm]	87.5	156.6	142.8	140.1	148.3	145.8	145.0
D90 [μm]	104.0	184.6	180.5	182.2	170.2	179.2	168.0
Rw	0.9	0.3	0.4	0.4	0.3	0.4	0.3

**Table 3:** Summary of Tukey’s test results for TS at 5 kN. *** *p* < 0.001; n.s., not significant.

Comparison Pair	Mean Difference	*p*-Value	Significance
2-1 vs. 1-2	1.06	<0.001	***
2-3 vs. 1-2	1.05	<0.001	***
1-1 vs. 1-2	0.86	<0.001	***
2-1 vs. 1-3	0.65	<0.001	***
2-3 vs. 1-3	0.64	<0.001	***
2-1 vs. 2-2	0.57	<0.001	***
2-3 vs. 2-2	0.56	<0.001	***
2-2 vs. 1-2	0.49	<0.001	***
1-1 vs. 1-3	0.44	<0.001	***
1-3 vs. 1-2	0.41	<0.001	***
1-1 vs. 2-2	0.37	<0.001	***
2-1 vs. 1-1	0.21	<0.001	***
2-3 vs. 1-1	0.19	<0.001	***
2-2 vs. 1-3	0.07	0.2164	n.s.
2-1 vs. 2-3	0.01	0.9987	n.s.

**Table 4:** Summary of Tukey’s test results for TS at 10 kN. *** *p* < 0.001; ** *p* < 0.01; * *p* < 0.05; n.s., not significant.

Comparison Pair	Mean Difference	*p*-Value	Significance
2-3 vs. 1-2	1.16	<0.001	***
2-1 vs. 1-2	1.02	<0.001	***
2-3 vs. 1-3	0.90	<0.001	***
1-1 vs. 1-2	0.82	<0.001	***
2-1 vs. 1-3	0.77	<0.001	***
2-2 vs. 1-2	0.72	<0.001	***
1-1 vs. 1-3	0.57	<0.001	***
2-2 vs. 1-3	0.47	<0.001	***
2-3 vs. 2-2	0.44	<0.001	***
2-3 vs. 1-1	0.34	<0.001	***
2-1 vs. 2-2	0.30	<0.001	***
1-3 vs. 1-2	0.26	<0.001	***
2-1 vs. 1-1	0.20	0.0022	**
2-3 vs. 2-1	0.14	0.0386	*
1-1 vs. 2-2	0.10	0.2077	n.s.

**Table 5:** Parameters of granules prepared with different granulation solvents and drying temperatures for each LXP sample.

Parameter	Original	1-1	1-2	1-3	2-1	2-2	2-3
BFE [mJ]	1850	737	818	2067	744	1156	2411
CBD [g/mL]	0.49	0.53	0.5	0.48	0.49	0.49	0.32
Cohesion [kPa]	0.85	0.5	0.42	0.5	0.58	0.42	0.68
FF	5.61	9.06	6.61	9.23	7.78	10.67	7.05
AIF [°]	35.04	41.84	39.22	42.54	43.4	41.36	39.69
γ(p) [mJ/m^2^]	9.44	9.96	3.09	12.4	24.06	21.92	29.84
γ(d) [mJ/m^2^]	8.33	16.08	22.13	16.82	9.98	8.19	6.64
SFE [mJ/m^2^]	17.78	26.03	25.21	29.22	34.04	30.11	36.48

**Table 6:** Correlation matrix of granule properties.

	BFE	CBD	Cohesion	FF	AIF	γ(p)	γ(d)	SFE	TS at 5 kN	TS at 10 kN
BFE	1	−0.8041	0.5539	−0.0627	−0.2215	0.4557	−0.3608	0.5462	0.1669	0.1574
CBD	−0.8041	1	−0.7843	0.4033	0.4434	−0.6862	0.5793	−0.7733	−0.4152	−0.4797
Cohesion	0.5539	−0.7843	1	−0.4333	0.044	0.7198	−0.5886	0.8368	0.815	0.7388
FF	−0.0627	0.4033	−0.4333	1	0.4933	0.0768	−0.2532	−0.1688	−0.019	0.0609
AIF	−0.2215	0.4434	0.044	0.4933	1	0.171	−0.1864	0.1359	0.4071	0.257
γ(p)	0.4557	−0.6862	0.7198	0.0768	0.171	1	−0.9738	0.9511	0.7584	0.854
γ(d)	−0.3608	0.5793	−0.5886	−0.2532	−0.1864	−0.9738	1	−0.8559	−0.7335	−0.8681
SFE	0.5462	−0.7733	0.8368	−0.1688	0.1359	0.9511	−0.8559	1	0.7279	0.7624
TS at 5 kN	0.1669	−0.4152	0.815	−0.019	0.4071	0.7584	−0.7335	0.7279	1	0.9528
TS at 10 kN	0.1574	−0.4797	0.7388	0.0609	0.257	0.854	−0.8681	0.7624	0.9528	1

**Table 7:** Loss on drying of each LXP sample.

Formulation	Original	1-1	1-2	1-3	2-1	2-2	2-3
Weight loss [%]	12.02	6.29	0.53	0.28	6.23	0.50	0.27

## Data Availability

The data presented in this study are available on request from the corresponding author.

## Supplementary Materials

The following supporting information can be downloaded at: https://www.mdpi.com/article/10.3390/pharmaceutics17040455/s1, Table S1: Analysis of variance (ANOVA) table for (a) TS at 5 kN, (b) TS at 10 kN.

## Abbreviations

The following abbreviations are used in this manuscript:

LXP	Loxoprofen Sodium
IB	Ibuprofen
APAP	Acetaminophen
OTC	over-the-counter
TS	Tensile Strength of Tablets
PCA	Principal Component Analysis
PC1	First Principal Component in Principal Component Analysis
PC2	Second Principal Component in Principal Component Analysis
SFE	Surface Free Energy
γ(d)	Dispersive Component
γ(p)	Polar Component
BFE	Basic Flow Energy
CBD	Conditioned Bulk Density
Cohesion	Shear Stress at Vertical Stress of 0 on the Destruction Envelope
FF	Flow Function: Defined as Maximum Principal Stress/Uniaxial Failure Stress
AIF	Angle of Internal Friction
