# Supplementary material for: Granulation Methods and the Mechanisms for Improving Hardness of Loxoprofen Sodium Hydrate-Containing Tablets
Source: Pharmaceutics. 2025 Apr 1;17(4):455. doi: 10.3390/pharmaceutics17040455 (PMC12030358; doi:10.3390/pharmaceutics17040455)
Supplement: Supplementary file 1 [file pharmaceutics-17-00455-s001.zip › pharmaceutics-3500052-supplementary.pdf]

Supplementary materials

Pharmaceutics

Granulation Methods and the Mechanisms for Improving Hardness of Loxoprofen Sodium Hydrate-Containing Tablets

Aya Kuwata [1,2,*], Agata Ishikawa [1], Tetsuo Ono [1] and Etsuo Yonemochi [2,3,*]

Table S1. Analysis of variance (ANOVA) table for (a) TS at 5kN, (b) TS at 10kN.

(a)

	DF[a]	MS[b]	F value	P value
X1: Solution	1	0.88	77.63	<0.001
X2: Dry temperature	2	0.80	70.28	<0.001
Error	14	0.01		
Total	17	0.16		

[a] Degree of freedom, [b] Mean square

(b)

	DF[a]	MS[b]	F value	P value
X1: Solution	1	1.68	55.22	<0.001
X2: Dry temperature	2	0.48	15.74	<0.001
Error	14	0.03		
Total	17	0.18		

[a] Degree of freedom, [b] Mean square
